# Alar Cartilage Hematoma: A Case Report

**DOI:** 10.7759/cureus.113204

**Published:** 2026-07-23

**Authors:** Ayesha Almarzooqi, Fay Alraddawi, Safeena Kherani, Hayat Adib

**Affiliations:** 1 Family Medicine, Sheikh Khalifa Medical City, Abu Dhabi, ARE; 2 Family Medicine, Al Mizhar Healthcare Center, Dubai Health, Dubai, ARE; 3 Family Medicine, Mohammed Bin Rashid University, Dubai, ARE; 4 Otolaryngology - Head and Neck Surgery, Mohammed Bin Rashid University, Dubai, ARE; 5 Otolaryngology - Head and Neck Surgery, Al Jalila Children's Specialty Hospital, Dubai, ARE; 6 Otolaryngology - Head and Neck Surgery, Dubai Health, Dubai, ARE

**Keywords:** alar hematoma, cartilage, drainage, maxillofacial trauma, nasal trauma

## Abstract

In maxillofacial trauma, the most affected structure is the nose due to its prominence on the face. Trauma to the nose can lead to fractures, whether bony or cartilaginous, soft tissue damage, or, less commonly, hematomas. The nasal septum is the typical site for hematoma development and is considered a surgical emergency. Another site for a hematoma is the alar cartilage; however, it is very rare. In this report, we present a rare case of an alar cartilage hematoma in a three-year-old female after facial trauma.

## Introduction

In maxillofacial trauma, the most commonly injured structure is the nose due to its prominent location on the face. Nasal fractures often coincide with nasal trauma, comprising a significant 48%-50% of all facial fractures [1]. These fractures can result from various mechanisms, such as motor vehicle collisions involving cyclists or pedestrians, sports-related injuries, including weightlifting, or accidents at home. Nasal trauma can lead to bony fractures, soft tissue injuries, or, less frequently, hematomas. Typically, nasal hematomas are located in the nasal septum. However, there have been a few cases documented in the alar cartilage.

Nasal hematomas occur when blood vessels within the perichondrium are torn. The collection of blood between the cartilage and the overlying intact perichondrium gives rise to a sub-perichondrial hematoma. This hematoma disrupts the blood supply to the cartilage and may lead to nasal cartilage necrosis and perforation. The hematoma can also evolve into an abscess that can spread further intracranially via the skull base [2]. A rare site of the hematoma is the alar cartilage, which seems to primarily affect children, with six of the already reported cases being in the pediatric population. The alar cartilage is C-shaped and closely approximated to the septum. It is composed of medial and lateral crura that join to form the nostril and nasal entrance [1]. When undiagnosed, nasal hematomas can lead to complications ranging from scarring and cartilage destruction to intracranial infections.

Hence, it is crucial to promptly detect and surgically drain the hematoma, in addition to providing antimicrobial coverage. In other words, timely diagnosis and management are essential to potentially avoid both aesthetic and functional complications associated with alar cartilage hematomas. In this report, we present a rare case of alar cartilage hematoma in a three-year-old female.

## Case presentation

A previously healthy three-year-old female was running when she tripped and hit her nose directly on the edge of a hard chair. There was no loss of consciousness or emesis post-injury. In the first hospital, she was given xylometazoline to treat epistaxis, paracetamol for analgesia, and ice packs to minimize swelling. Thereafter, she presented to the Emergency Department of our tertiary care pediatric hospital with post-traumatic epistaxis and nasal congestion.

The otolaryngology team was consulted. Examination revealed visible swelling and bruising on the bridge of the nose. Additionally, there was a small hematoma on the lateral side of the left nasal cavity at the caudal end corresponding to the location of the ala. The edematous lateral nostril mucosa was red to purple in color, covered with blood crust, and had minimal oozing of blood. No bony deformity was noted. Anterior rhinoscopy of the left septum was not possible because of the obstructive internal left alar swelling and tenderness with manipulation. The right side of the septum only had scant blood with no apparent hematoma and normal right nasal alar tissue. An X-ray had been completed and did not reveal any nasal fracture. Blood tests showed a normal blood count and coagulation profile.

**Figure 1:**
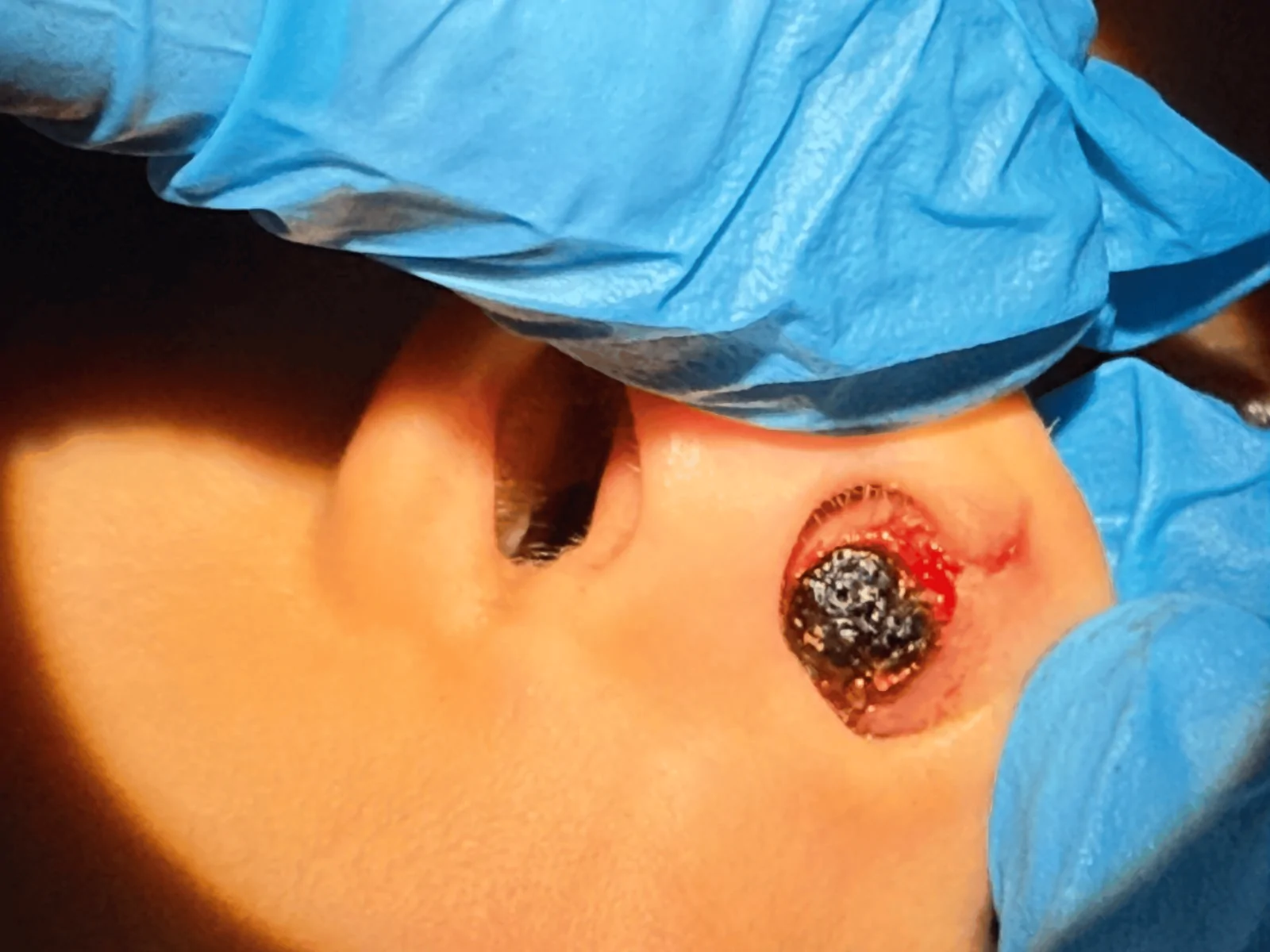
Left alar hematoma

The patient was taken to the operating theatre for incision and drainage of the alar hematoma within 24 hours of the injury. Using a zero-degree nasal endoscope, bilateral nasal passageways were examined to the level of the choana. A lateral bulge suggestive of a hematoma covered with a crust was observed anteriorly at the level of the left alar cartilage (Figure 1). An incision was made using an 11-blade along the most convex portion of the lesion, releasing blood and consequently reducing the swelling significantly. To prevent recurrence of the hematoma, a Merocel pack coated with fucidic acid antimicrobial ointment was inserted in the patient's left nasal cavity, ensuring appropriate anterior coverage by the ala. Systemic antibiotics were prescribed. Postoperatively, the patient was monitored overnight and showed no signs of recurrence, fever, bleeding, swelling, or shortness of breath. Following the observation, she was discharged on oral antibiotics for a total of a two-week course. During both her one-week and two-week follow-up appointments, there were no symptoms of nasal bleeding, congestion, or obstruction, and anterior rhinoscopy did not reveal any evidence of infection or recollection.

## Discussion

Alar hematoma refers to a blood collection between the alar cartilage and the overlying intact perichondrium [1]. Children seem to be more likely to sustain a septal nasal injury, likely due to the presence of softer septal cartilage, which is more prone to buckling [3]. Nasal alar hematoma is very uncommon and was first described in the literature in 1994 [4]. The mechanism of alar cartilage hematoma formation is nasal trauma, and it has been observed primarily in the pediatric population [4]. In the above case, the hematoma developed in the absence of any predisposing coagulopathy. In children, the nasal cartilage tends to be more vascularized and less ossified than in adults. The cartilage tissue has a weak and loosely adherent mucoperichondrium [5]. As such, children may thus be more prone to develop alar cartilage hematoma than adults [6].

Cartilage tissue is an avascular structure that acquires oxygen from the perichondrial capillaries. A developing hematoma would prevent the alar cartilage from receiving sufficient glucose and oxygen [7]. A hematoma left untreated can become infected, developing into an abscess and possibly resulting in severe complications. Such complications can range from severe aesthetic deformities to intracranial sequelae leading to death [8].

Compared to an alar hematoma, which is usually unilateral, a septal hematoma is more likely to be bilateral, especially when the trauma is severe. The process involves the rupture of submucosal vessels, which causes a collection of blood between the nasal septal cartilage and the perichondrium. If left untreated, infection of the area can occur within 72 hours, possibly leading to a septal perforation [2]. Both hematomas are vague in symptoms and require awareness of the condition and a high level of suspicion. Furthermore, urgent incision and drainage are needed in both [2].

This presentation of alar cartilage hematoma has yet to be studied, and due to the sparse number of cases, there are no proper guidelines on the management of nasal alar hematomas. Considering its close approximation to the septum and being of similar tissue quality, it stands to reason that the correct assessment and management of nasal alar hematomas are also crucial to prevent complications. Early diagnosis will reduce the risk of abscess formation and nasal deformity [3]. The patient in this case underwent hematoma evacuation within 24 hours of the injury, and subsequent monitoring revealed no discernible functional or cosmetic complications.

## Conclusions

Sub-perichondral hematomas of the alar cartilage are rare but can be encountered following nasal trauma. Just like septal hematomas, this case highlights the significance of early detection and treatment to avert potential complications. Although alar cartilage hematomas post-nasal injury are infrequent, this case emphasizes the importance of a thorough assessment of facial and nasal injuries, including the alar rims, to facilitate accurate diagnosis and appropriate management so as to mitigate adverse outcomes.
